# Immunization Efficacy of a Commercialized Inactivated *Mycoplasma bovis* (Strain HM) Vaccine in Calves at a Large-Scale Dairy Farm in Yancheng, Jiangsu Province

**DOI:** 10.3390/vetsci13101026

**Published:** 2026-09-28

**Authors:** Yingjian Li, Ruihua Zhang, Yiquan Jin, Lei Wang, Kai Huang, Yongzhan Bao, Xuejian Zhao, Jingyan Zhang, Zhengzhong Luo, Zhiting Guo, Xiaorong Lu, Zhiyong Hao, Kang Zhang, Jianxi Li

**Affiliations:** 1 Lanzhou Institute of Husbandry and Pharmaceutical Sciences, Chinese Academy of Agricultural Sciences, Lanzhou 730050, China; o264546465@163.com (Y.L.);; 2 College of Traditional Chinese Veterinary Medicine, Hebei Agricultural University, Baoding 071001, China; 3 Bright Farming Co., Ltd., Shanghai 201103, China; 4 College of Animal Science and Technology, Ningxia University, Yinchuan 750021, China

**Keywords:** bovine respiratory disease, inactivated *Mycoplasma bovis* vaccine, calves, large-scale dairy farms, protective efficacy

## Abstract

*Mycoplasma bovis* (*M. bovis*)-positive clinical Bovine Respiratory Disease (BRD) seriously threatens the survival and growth of calves. Against the global backdrop of escalating antimicrobial resistance, effective vaccines represent a promising strategy for the prevention and control of *M. bovis*-positive clinical BRD. This study aimed to characterize the epidemiological features of BRD on a large-scale dairy farm in Yancheng, Jiangsu Province, and to assess the efficacy of an inactivated *M. bovis* strain HM vaccine using a randomized, double-blind, controlled field trial conducted during the high-incidence season. In total, 200 healthy 3–4-week-old Holstein calves seronegative for *M. bovis*-specific antibodies were randomly allocated to either a vaccine group or a control group. Calves in the vaccine group received two intramuscular vaccine doses on day 0 and day 14, while control calves received an equal volume of placebo at identical time points. The entire trial spanned 194 days. A comprehensive assessment was carried out, including vaccine tolerability, growth performance, serum antibody and cytokine profiles, BRD incidence, infection rates of common pathogens, and vaccine protective efficacy. The results showed that the farm-level annual BRD incidence was 9.27%, consistent with typical temporal–spatial-herd distribution patterns. Calves exhibited good tolerability after vaccination, and the vaccine had no adverse effect on body-weight gain. Importantly, vaccination markedly elevated serum specific antibody levels, seropositivity rates, and concentrations of inflammatory cytokines including IL-4, IL-6, and IL-10 (*p* < 0.05). Moreover, vaccination reduced the risk of *M. bovis*-positive clinical BRD by 61% and decreased infection risks for several respiratory pathogens. Collectively, this inactivated vaccine demonstrated satisfactory tolerability and protective effects, providing critical technical evidence for the prevention and control of *M. bovis*-positive clinical BRD in dairy farms.

## Simple Summary

Bovine Respiratory Disease (BRD) caused by *Mycoplasma bovis* (*M. bovis*) infection results in substantial economic losses to the livestock industry. Therefore, it is urgent to develop effective vaccines to prevent and control *M. bovis*-positive clinical BRD. To systematically evaluate the efficacy of a commercially available inactivated *M. bovis* vaccine (strain HM) in the field, a randomized, double-blind, controlled field trial at a large-scale dairy farm in Jiangsu Province was conducted in this study. The results indicated that the vaccine was well-tolerated. Moreover, a booster immunization was effective in inducing specific immune responses, reaching high protective efficacy (61%) against *M. bovis*-positive clinical BRD. Furthermore, the vaccine significantly reduced the incidence of bovine respiratory disease (BRD) and the infection risks of certain pathogens. These findings demonstrated that the vaccine afforded excellent protection against *M. bovis*-positive clinical BRD in calves. This study offers important technical support for controlling *M. bovis*-positive clinical BRD in farms.

## 1. Introduction

Bovine respiratory disease (BRD) is a complex respiratory syndrome caused by multiple factors [1]. The global prevalence of BRD varies greatly owing to numerous factors including geography, season, farming systems, and stocking densities [1,2]. *Mycoplasmas* are closely associated with BRD, mainly including *Mycoplasma bovis* (*M. bovis*), *Mycoplasma bovirhinis* (*M. bovirhinis*), and *Mycoplasma dispar* (*M. dispar*), among which *M. bovis* is the most prevalent in cattle herds. Cattle with *M. bovis*-positive clinical BRD exhibit respiratory clinical signs such as coughing and serous nasal discharge. Without prompt treatment, the disease can progress rapidly, with clinical manifestations including high fever, tachypnea, and lethargy [3]. When infection further invades the pulmonary parenchyma and induces characteristic lesions, it develops into *M. bovis*-attributable pneumonia, which is pathologically characterized by bronchial interstitial pneumonia, bronchial-associated lymphoid tissue hyperplasia, and caseous necrosis [4,5,6]. *Mycoplasma bovis*, a member of the class Mollicutes, is a cell-wall-less microorganism. Existing studies have indicated that it is not merely a secondary opportunistic pathogen. In some endemic scenarios, it can act as a primary or dominant pathogen of bovine respiratory disease (BRD), inflicting multiple-faceted hazards on the global beef and dairy cattle-raising industries [1,7].

Currently, antibiotic supplementation in feed is the primary approach for controlling *M. bovis* infections in many countries [8]. However, the lack of cell wall makes *M. bovis* naturally resistant to β-lactam antibiotics. Furthermore, long-term antibiotic abuse has given rise to antibiotic resistance, which renders antibiotics less effective [9,10]. In China, multiple studies have reported that clinically isolated *M. bovis* showed a multidrug-resistant phenotype [11,12,13]. With the implementation of the Draft Global Action Plan on Antimicrobial Resistance 2026–2036, developing safe and effective vaccines has become a rational strategy for controlling *M. bovis*-positive clinical BRD. Prior to 2021, commercially available *M. bovis* vaccines existed only in the United States, and these vaccines exhibited highly variable protective efficacy. In addition, several inactivated *M. bovis* vaccines remained under research and validation [14,15,16].

The inactivated *M. bovis* (strain HM) vaccine was approved for marketing in China in 2025. However, its protective efficacy under mixed-infection conditions in the field had not yet been investigated. Moreover, whether the immunization regimen was suitable for calves had not been determined. Therefore, with permission from the vaccine manufacturer, we performed a randomized, double-blind, controlled field trial at a large-scale dairy farm in Yancheng, Jiangsu Province. The trial enrolled 3–4-week-old healthy Holstein calves that were seronegative for *M. bovis*. The clinical efficacy of this inactivated vaccine was systematically evaluated by assessing vaccine tolerability, calf growth parameters, serum antibody levels, and protective effects. The findings of this study will provide critical technical evidence for the prevention and control of *M. bovis*-positive BRD in dairy farms.

## 2. Materials and Methods

### 2.1. Vaccine

The inactivated *M. bovis* (strain HM) vaccine (Veterinary Drug Approval No. 310013031) was an oil-emulsion vaccine containing inactivated *M. bovis* strain HM. The viable bacterial count was 1.56 × 10^9^ CFU per 2-mL dose before inactivation. It was developed by Tecon Biopharmaceutical Co., Ltd. (Urumqi, China).

### 2.2. Main Reagents and Instruments

The *M. bovis* antibody ELISA kit (Cat. No. E023) was purchased from Beijing Biaochi Zehui Biotechnology Co., Ltd. (Beijing, China). The ELISA kits for detecting bovine interleukin-4 (IL-4) (Cat. No. ml002501), interleukin-6 (IL-6) (Cat. No. ml064296), interleukin-10 (IL-10) (Cat. No. ml002476), tumor necrosis factor-α (TNF-α) (Cat. No. ml077389), and interferon-γ (IFN-γ) (Cat. No. ml002465) were purchased from Shanghai Enzyme-linked Biotechnology Co., Ltd. (Shanghai, China). The animal pathogens nucleic acid extraction test kit (Cat. No. AE3EK010) was purchased from Shenzhen Anieasy Biotechnology Co., Ltd. (Shenzhen, China). The multi-8 bovine respiratory disease syndrome nucleic acid test kit (Cat. No. YRMBP7075) was purchased from Shenzhen Bioeasy Biotechnology Co., Ltd. (Shenzhen, China).

The high-speed centrifuge (Model: 5430 R) was purchased from Eppendorf AG (Hamburg, Germany); the laminar-flow clean bench (Model: SCB-920) was purchased from Beijing Donglian Har Instrument Manufacture Co., Ltd. (Beijing, China); the metal dry-bath heater (Model: HB120-S) was purchased from Beijing Sinoport Equipment Co., Ltd. (Beijing, China); the microplate thermoshaker (Model: ST70-2) was purchased from Hangzhou Miu Instruments Co., Ltd. (Hangzhou, China); the microplate reader (Model: FLX800T) was purchased from BioTek Instruments, Inc. (Winooski, VT, USA); the real-time qPCR system (Model: YR 8000) was purchased from Shenzhen Bioeasy Biotechnology Co., Ltd. (Shenzhen, China); the intelligent body-weight and body-size measurement system for cattle (Model: BMCZTC-5) was purchased from Golden Grassland Intelligent Technology (Shanghai) Co., Ltd. (Shanghai, China).

### 2.3. Experimental Site and Animals

This study was conducted in December 2025 at a large-scale dairy farm in Yancheng, Jiangsu Province (Figure 1). Sera were collected from 3–4-week-old calves and tested for *M. bovis*-specific antibodies; the antibody-positive rate was approximately 10%. A total of 200 healthy 3–4-week-old Holstein calves without congenital defects, which tested seronegative for *M. bovis*-specific antibodies, were randomly selected. The barns were thoroughly cleaned and disinfected prior to the trial. All enrolled calves were weaned at 60 days of age.

### 2.4. Grouping and Immunization

Thirty-two calves were randomly selected from the 200 calves (24.19 ± 1.53 days of age, body weight: 62.66 ± 3.59 kg) and divided into vaccine group A (16 calves) and control group A (16 calves). The remaining 168 calves were randomly divided into vaccine group B (84 calves) and control group B (84 calves). Simple randomization was used for group allocation, and the random sequence was generated in advance by an investigator using the built-in random-number generator of SPSS Statistics 27.0. For allocation concealment, group-assignment information was sealed in sequentially numbered, opaque envelopes, which were securely stored by the vaccine manufacturer and inaccessible to researchers, farm veterinarians, and animal caretakers before all data collection was completed. Subgroup A was set for immunodynamic monitoring, which required continuous jugular vein blood sampling and weight measurements. These procedures could cause stress to the calves and increase the risk of pathogen infection. Therefore, the sample size for subgroup A was relatively small. Subgroup B was set for testing the field efficacy of the vaccine, which was subjected only to routine rearing observations and nasal swab collection from calves diagnosed with BRD to identify the causative pathogens. A large sample size for this subgroup ensured more reliable statistics for *M. bovis*-positive clinical BRD incidence rates. Following random allocation, calves from the two groups were housed in separate individual hutches and spatially interspersed across the rearing area. This interspersed spatial layout minimized confounding bias originating from spatial heterogeneity of pathogen exposure across different locations of the farm. All trial-related operational procedures were standardized and identical between groups, including the timing of vaccination, antimicrobial treatment protocols, daily frequency of clinical surveillance, clinical scoring criteria for BRD, and pathogen-sampling rules. Vaccination records, antimicrobial administration logs, clinical scoring sheets, and sampling logs were archived in the farm’s electronic management system. These archived records were reviewed after database lock to verify the absence of systematic between-group differences in management practices. Therefore, the design supports attribution of the observed reduction in BRD incidence to vaccination rather than spatial or management confounders.

Calves in the vaccine groups (A + B) were immunized with 2 mL of the inactivated *M. bovis* (strain HM) vaccine via intramuscular injection in the neck, followed by a booster dose (2 mL) on day 14 as recommended by the manufacturer. Calves in the control groups (A + B) received an equal volume of placebo at the same time points. The placebo was a blank adjuvant emulsion. The adjuvant components, emulsification process, solvent, and appearance were identical to those of the vaccine with the sole difference of the absence of the inactivated *M. bovis* HM strain antigen. Both the vaccine and placebo were prepared and blinded by the vaccine manufacturer. All study personnel, including farm staff, veterinarians performing clinical scoring, laboratory technicians processing samples, and researchers conducting data analysis, remained blinded to group assignment throughout sample processing, laboratory testing, and data analysis. Pre-defined emergency unblinding criteria were applied: unblinding was only permitted if a calf experienced a life-threatening adverse event requiring immediate clinical intervention, though no emergency unblinding occurred in this trial. The group-decoding information was released by the manufacturer only after data collection was finished and the database was locked. All immunizations were administered by the same veterinarian, and immunological parameters were monitored over a 60-day period. According to the vaccine manufacturer’s instructions, the expected duration of protective immunity after booster immunization was six months; therefore, the overall field observation period was set to 194 days. The study design is shown in Figure 2.

### 2.5. Clinical Scoring Criteria for BRD

In this study, the BRD3 scoring system developed by Love et al. was used to assess BRD in pre-weaned calves. Calves with a total score ≥ 5 points were defined as positive for BRD [17]. Model 1, the comprehensive BRD scoring model developed by Maier et al., was used to assess BRD in post-weaned calves. Calves obtaining a total score ≥ 2 points were defined as BRD-suspect; suspect calves were classified as confirmed BRD cases if their rectal temperature was ≥39.2 °C [18]. Clinical BRD sign scoring was performed by the same veterinarian.

### 2.6. Sample Collection

Serum samples were collected on day 0, 7, 14, 21, 28, and 60. A total of 5 mL of blood was collected via the jugular vein. The blood was then incubated at 37 °C for 1 h to allow clotting. After centrifugation at 4 °C and 3000 rpm for 10 min, serum was harvested and transferred into 1.5-mL cryovials. Nasal swabs were collected from all calves diagnosed with BRD and placed into cryovials. All samples were stored at −80 °C until analysis.

### 2.7. Analysis of BRD Epidemiological Characteristics

The information about cattle population and BRD cases from December 2024 to November 2025 was exported from the UniDairy Farm Management System (Beijing Hemu Xingbang Network Technology Co., Ltd., Beijing, China), including onset date, age, physiological stage, barn number, and treatment outcome. The number of BRD cases across different seasons (spring: March–May 2025; summer: June–August; autumn: September–November; winter: December 2024–February 2025) and the incidence rates in each month were calculated to analyze the temporal distribution of BRD. The number of BRD cases in different barns was calculated to analyze the spatial distribution of BRD. The incidence rates across different physiological stages (suckling calves, weaned calves, growing heifers, lactating cows) and by age were calculated to analyze the distribution of BRD within the herd. The duration of treatment for each case was collected and summarized to calculate the cure rate.

### 2.8. Tolerability and Growth Performance Evaluation

The tolerability of the vaccine was evaluated for all calves by monitoring for abnormalities in mental status, feed and water intake, and body temperature for 7 consecutive days following each vaccination. Rectal temperature was measured daily. Particular attention was paid to local reactions at the injection site, such as redness, swelling, induration, or suppuration.

The growth performance of calves was evaluated based on average daily gain (ADG). All calves in subgroup A (both vaccine and control) were weighed prior to morning feeding on day 0, 14, 28, and 60. Body weights at different time points were recorded, and the ADG for each phase was calculated according to Equation (1):

(1) ADG = (Weight at the end of the phase − Weight at the beginning of the phase)/Number of days in the phase

### 2.9. Evaluation of the Immune Responses

The relative antibody levels were detected by indirect ELISA. The optical density (OD) values of serum samples from subgroup A (both vaccine and control) at day 0, 14, 21, 28, and 60 were obtained, from which the relative antibody levels were calculated using Equation (2):

(2) S/P value = (OD_450_ nm value of test serum − Average OD_450_ nm value of negative control serum)/(Average OD_450_ nm value of positive control serum − Average OD_450_ nm value of negative control serum)

According to the instructions, sample-to-positive ratio (S/P) value ≥ 0.3 was defined as positive, and S/P value < 0.3 was defined as negative. The positivity rate was calculated using Equation (3):

(3) Antibody positive rate = Number of positive calves/Total number of calves in subgroup A (n = 16) × 100%

All serum samples were tested using the same assay kit under the same experimental conditions. The indirect ELISA reflects the antibody levels in a sample: a higher S/P value indicates a higher level of specific antibodies in the sample. Therefore, the S/P value was used in this study to assess antibody levels.

The sandwich ELISA was used to determine the levels of IL-4, IL-6, IL-10, TNF-α, and IFN-γ in the cattle sera from subgroup A (both vaccine and control) at day 0, 14, 21, 28, and 60. All procedures were performed strictly according to the manufacturer’s instructions.

### 2.10. Evaluation of Protective Effects

In accordance with Section 2.5 (Clinical Scoring Criteria for BRD), the number of BRD cases in calves of the vaccine group (A + B) and control group (A + B) was counted, and the BRD incidence was calculated. The common pathogens associated with BRD were detected via reverse transcription quantitative polymerase chain reaction (RT-qPCR). Nucleic acids (DNA/RNA) were first extracted from nasal swab samples of sick calves according to the nucleic acid extraction kit instructions. Next, a 25 μL reaction mixture was prepared using the multi-8 bovine respiratory disease syndrome nucleic acid test kit (Table 1). RT-qPCR was performed as previously described [19], with thermal conditions as follows: reverse transcription at 45 °C for 10 min (1 cycle), pre-denaturation at 95 °C for 3 min (1 cycle), followed by 40 cycles of denaturation at 95 °C for 10 s and combined annealing/extension at 60 °C for 30 s, with fluorescence signals collected at each cycle. Pathogen detection results were interpreted following the manufacturer’s kit instructions.

Calves presenting clinical signs of bovine respiratory disease (BRD) and with positive *M. bovis* PCR results in respiratory samples were defined as *M. bovis*-positive clinical BRD cases. Relative risk (RR), vaccine efficacy (VE), absolute risk reduction (ARR), and number needed to vaccinate (NNV) were calculated according to the published literature to evaluate the protective efficacy of the vaccine against *M. bovis*-positive clinical BRD [20,21]:

(4) RR = Incidence rate in the vaccine group (A + B)/Incidence rate in the control group (A + B)

(5) VE = (1 − RR) × 100%

(6) ARR = Incidence rate in the control group (A + B) − Incidence rate in the vaccine group (A + B)

(7) NNV = 1/ARR

### 2.11. Statistical Analysis

Experimental data were preliminarily processed using Microsoft Excel 2016 (Microsoft Corp., Redmond, WA, USA), and results were expressed as mean ± standard deviation (SD). Statistical analysis was performed using SPSS Statistics 27.0 (IBM Corp., Armonk, NY, USA). The Shapiro–Wilk test was used to assess the normality of data, and Levene’s test was applied to evaluate homogeneity of variance. Comparisons among multiple groups were conducted using one-way ANOVA, while comparisons between two groups were performed using the independent samples *t*-test. For categorical data of two independent groups, the chi-square (*χ*^2^) test was used; when theoretical frequencies were insufficient, Fisher’s exact test was adopted for supplementary analysis. A *p*-value < 0.05 indicated a significant difference, *p* < 0.01 indicated a highly significant difference, and *p* < 0.001 indicated an extremely significant difference. GraphPad Prism 8 (GraphPad Software Inc., San Diego, CA, USA) was used for graphing.

## 3. Results

### 3.1. Incidence of BRD

The incidence of BRD on the dairy farm was analyzed as shown in Table 2. During the period from December 2024 to November 2025, a total of 714 calves developed BRD, corresponding to an average annual incidence rate of 9.27%. The number of cases in spring, summer, autumn, and winter was 237, 130, 136, and 211, respectively.

### 3.2. Analysis of BRD Epidemiological Characteristics

The incidence rate of BRD kept rising from December 2024 to April 2025 (reaching the peak), followed by a gradual decline, and remained at a low level from September to November 2025 as shown in Figure 3a. There were significant differences in the number of BRD cases across different barns as shown in Figure 3b, and most cases occurred in Barns 90–120. Notably, the BRD cases among suckling calves (589), weaned calves (90), growing heifers (34), and lactating cows (1) varied substantially as shown in Figure 3c. Excluding one lactating cow that developed BRD at 1092 days of age, the average age at onset was 55 days. The peak incidence was observed in suckling calves aged 20–60 days (Figure 3d). With the exception of 15 cattle that were treated for more than 30 days, the duration of treatment for BRD cases ranged from 5 to 7 days (average of 6.7 days) as shown in Figure 3e. The cure rate for BRD cases was 98% and the remaining 2% of cases were not cured (Figure 3f).

### 3.3. Results of Tolerability and Growth Performance Evaluation

During the trial, one calf in subgroup B died of mechanical asphyxiation due to pen-trapping. This death was unrelated to vaccination or trial-related procedures. Upon global unblinding at trial completion, the animal was confirmed to belong to vaccine subgroup B. Following exclusion of this trial-lost animal according to the pre-specified Statistical Analysis Plan, the pooled vaccine group (A + B) and pooled control group (A + B) finally included 99 and 100 calves, respectively.

As shown in Table 3, calves in both vaccine groups and the control groups exhibited normal mental status, feed and water intake, and excretion. The body temperatures remained within the healthy ranges throughout the trial. No abnormal reactions such as redness, swelling, induration, or inflammation occurred at the injection sites in all calves. No death happened due to vaccination.

In terms of body weight, there was no significant difference between vaccine group A and control group A (*p* > 0.05) at 0, 14, 28, and 60 days as shown in Table 4 and Figure 4. Moreover, there was no significant difference in ADG between vaccine group A and control group A at each time point (*p* > 0.05).

### 3.4. Evaluation of the Immune Response

#### 3.4.1. Determination of Relative Antibody Levels in Serum and Calculation of the Positive Rate

The relative antibody levels of vaccine group A at days 0, 7, and 14 were similar without significant changes (*p* > 0.05), which showed no significant difference compared with control group A (*p* > 0.05) as shown in Figure 5. In contrast, the relative antibody levels in vaccine group A significantly increased at days 21 and 28 (*p* < 0.05), which were extremely significantly higher than those in control group A (*p* < 0.001). At day 60, the relative antibody levels in vaccine group A significantly decreased (*p* < 0.05), but were still significantly higher than those in control group A (*p* < 0.05). The relative antibody levels in control group A showed no significant differences among days 0, 7, 14, 21, and 28 (*p* > 0.05), but increased significantly at day 60 (*p* < 0.05).

As shown in Figure 6, the antibody positivity rates in both vaccine group A and control group A were 0% at days 0 and 7. On day 14, the positivity rates in vaccine group A and control group A were 18.75% (3/16) and 0%, respectively, which did not show significant differences (*p* > 0.05). On day 21, the antibody positivity rates in vaccine group A were 68.75% (11/16), which were highly significantly higher than those in control group A (0%) (*p* < 0.01). On day 28, the antibody positivity rates in vaccine group A rose to 81.25% (13/16), whereas those in control group A remained at 0%, showing an extremely significant difference (*p* < 0.001). On day 60, the antibody positivity rates in vaccine group A remained at 81.25% (13/16), while those in control group A rose to 25.00% (4/16) with a highly significant difference between the groups (*p* < 0.01).

#### 3.4.2. Serum Cytokine Assessment

There were no significant differences in IL-4 levels between the two groups at days 0, 7, 14, and 21 (*p* > 0.05) as shown in Figure 7a. The IL-4 levels in vaccine group A were significantly elevated (*p* < 0.05) on day 28, which were extremely significantly higher than those in control group A (*p* < 0.001). Furthermore, IL-4 levels decreased significantly by day 60 (*p* < 0.05), but there was no significant difference compared with control group A (*p* > 0.05).

The IL-6 levels showed no significant differences between the two groups (*p* > 0.05) at days 0, 7, 14, and 21, as shown in Figure 7b. IL-6 levels in vaccine group A on day 28 were significantly elevated (*p* < 0.05), which were extremely significantly higher than those in control group A (*p* < 0.001). On day 60, IL-6 levels in vaccine group A were significantly reduced (*p* < 0.05), but there was no significant difference compared with control group A (*p* > 0.05).

In terms of IL-10 levels, there were no significant differences in vaccine group A at days 0, 7, and 14 (*p* > 0.05), nor were there significant differences between vaccine group A and control group A (*p* > 0.05) as shown in Figure 7c. The IL-10 levels in vaccine group A gradually increased from day 14 onward, which were extremely significantly higher than those in control group A (*p* < 0.001) at days 21 and 60. In contrast, the IL-10 levels in control group A rose significantly on day 28 (*p* < 0.05) and showed no significant difference compared with vaccine group A (*p* > 0.05).

The TNF-α levels in vaccine group A were significantly higher than those in control group A on day 7 (*p* < 0.01) as shown in Figure 7d. TNF-α levels in vaccine group A rose significantly (*p* < 0.05) at day 60; however, no significant difference was observed versus the control group A (*p* > 0.05). There were no significant differences in TNF-α levels between the two groups at days 0, 14, 21, and 28 (*p* > 0.05).

There were no significant differences in IFN-γ levels between vaccine group A and control group A at days 0, 7, 14, 21, and 28 (*p* > 0.05) as shown in Figure 7e. IFN-γ levels in vaccine group A were extremely significantly higher than those in control group A on day 60 (*p* < 0.001).

### 3.5. Results of Evaluation of Protective Effects

As shown in Table 5, during the overall observation period of 0–194 days, the BRD incidence was 21.21% (21/99) in the vaccine group versus 36.00% (36/100) in the control group, showing a statistically significant reduction in the vaccine group (*p* < 0.05). No statistically significant inter-group differences in BRD incidence were observed for the pre-weaning (0–60 d) and post-weaning (61–120 d and 121–194 d) periods (*p* > 0.05). The BRD case-fatality rate was 0% in both groups throughout the 0–194-day trial.

As shown in Table 6 and Figure 8, all BRD calves in both groups were negative for BVDV, BPIV3, BRSV, and IBRV. Moreover, the infection rates of BCoV, *P. multocida*, *M. haemolytica*, and *M. bovis* in the vaccine group were 19.05%, 42.86%, 0%, and 61.90%, respectively, while those in the control group were 25.00%, 80.56%, 16.66%, and 91.67%, respectively. The infection rates for *P. multocida* and *M. bovis* in the vaccine group were both significantly lower than those in the control group (*p* < 0.05).

As shown in Table 7, over the entire 0–194-d trial, *M. bovis*-positive clinical BRD incidence was significantly lower in the vaccine group (13.13%) than in the control group (33.00%) (*p* < 0.001). The RR, VE, ARR, and NNV were 0.39, 61%, 19.87%, and 5.03, respectively (Table 8).

## 4. Discussion

The investigation of the BRD incidence on the large-scale dairy farm in Yancheng, Jiangsu Province, was conducted before the field trial, which showed that the average annual incidence of BRD was 9.27%. The data fell within the global prevalence range of 2.1% to 20.2% for BRD [2]. The unique and highly variable epidemiological patterns of BRD contributed to significant losses [2]. Therefore, the epidemiological characteristics of BRD at this dairy farm were further analyzed. Winter and spring were found to be peak seasons for BRD, which differed from the findings by Closs et al. [22]. We speculated that this discrepancy was related to regional climatic differences. Studies have shown that BRD usually occurs in cold seasons, when poor ventilation, high stocking densities, and cold stress facilitate BRD [23,24]. Sudden changes of temperature and large diurnal temperature fluctuations in winter and spring lead to a high incidence of BRD in China, which is consistent with the findings of Zhou et al. [25]. The BRD occurred primarily in suckling calves between 20 and 60 days of age, which is consistent with the findings of O’Donoghue S et al. [2]. During this period, calves are in the “critical window”, making them the most vulnerable to infections [26,27]. There were significant differences in the number of BRD cases across different barns. The majority of BRD occurred in Barns 90–120. As revealed by field investigation, these barns were primarily used for calf rearing. Moreover, these barns generally had issues such as excessive stocking density, inappropriate ventilation design, delayed manure removal, and insufficient implementation of disinfection measures, which have been proven to trigger BRD [28]. It is noteworthy that the average treatment duration for BRD was 6.7 days with a cure rate as high as 98%. The overall treatment efficacy was satisfactory. However, the existing literature indicates that improvements in respiratory clinical sign scores occur significantly earlier than the complete resolution of pulmonary consolidations. Veterinarians who rely solely on clinical signs to confirm recovery are at risk of misjudgment; therefore, continuous follow-up combined with lung ultrasonography is required to evaluate the actual therapeutic effect [29]. In addition, information regarding continuous pathogen monitoring and antibiotic susceptibility is limited because this farm does not routinely conduct pathogen testing for BRD. In the future, regular nucleic acid testing for respiratory pathogens, bacteria isolation, and antibiotic susceptibility testing should be conducted. Because *M. bovis* tends to form biofilms in the respiratory tracts, conventional short-course antimicrobial regimens may lead to incomplete treatment and recurrent infections. Therefore, the standard treatment course is recommended to be extended to 7–14 days and discontinuation of medication immediately upon sign relief is strictly prohibited [1,2].

Given that winter and spring are the peak seasons of BRD in calves and the BRD incidence increases from December, we decided to implement the vaccination in early December in this field trial. Tolerability constitutes a key factor for vaccine safety evaluation. The results of this study showed that calves vaccinated with the inactivated *M. bovis* (strain HM) vaccine did not exhibit systemic abnormal signs, local adverse reactions at the injection site, or vaccine-related deaths. This tolerability profile of the *M. bovis* vaccine has also been confirmed in other studies [30,31]. However, some studies have reported adverse reactions [15,16]. Furthermore, no significant differences were observed in body weight or ADG, which is consistent with field trials of other *M. bovis* vaccines [30,31]. The above findings confirmed that this vaccine exhibited excellent tolerability and strong production compatibility in the population of 3–4-week-old calves, meeting the clinical requirements for mass vaccination of calves in large-scale farms.

The immune response is a core indicator to evaluate the immunogenicity of vaccines. This study found that 10% of calves at 2–4-week-old were *M. bovis* antibody-positive, suggesting that maternal antibodies in this population had waned as previously reported [26]. To avoid the interference with maternal antibodies on the immune responses elicited by the vaccine [32], all calves involved in the trial were rigorously screened as negative for *M. bovis*-specific antibodies. Unlike the findings of Dudek et al., who reported rapid antibody elevation within 0–14 days after primary immunization with inactivated *M. bovis* vaccine [33], the inactivated *M. bovis* (strain HM) vaccine in this study did not elicit a marked antibody response in the early period. However, both the antibody levels and the seropositivity rate induced by the vaccine were significantly higher than those in the control group after a booster dose. Furthermore, the long-term maintenance of high antibody levels was comparable to that of the commercial vaccine Myco-B ONE DOSE™ [34]. These results indicated that the inactivated *M. bovis* (strain HM) vaccine could elicit strong humoral immune responses with a booster dose. At day 60, antibody levels in the vaccine group significantly decreased, but the seropositivity rate did not decline, confirming that there was no absolute correlation between antibody levels and seroconversion rates [35]. These findings provide direct guidance for establishing field vaccination protocols for *M. bovis* in calves and for interpreting antibody monitoring results, thereby avoiding misjudgments in assessing vaccination efficacy based solely on a single antibody titer measurement.

It is generally believed that *M. bovis* is an extracellular pathogen that mainly induces a Th2-type immune response to promote antibody production [36]. IL-4, a key Th2 cytokine, can effectively promote and maintain the Th2-type immune responses [37]. Both IL-4 and IL-6 are associated with B cell activation and contribute to antibody generation [38,39]. However, studies have shown that *M. bovis* can enter and survive in host cells, inducing Th1-skewing immune responses [40,41]. Th1 cells primarily combat intracellular pathogens and enhance the production of IFN-γ and TNF-α [41,42]. IL-10 is an anti-inflammatory cytokine that suppresses excessive inflammatory responses [43]. Some inactivated *M. bovis* vaccines elicit incomplete immune responses, with no significant differences in various cytokine levels compared with the control group [14]. In contrast, IL-4 and IL-6 levels were significantly elevated between day 21 and 28 in this study, suggestive of a serum-level Th2-like response phenotype in calves. On the other hand, TNF-α and IFN-γ levels increased significantly between day 28 and 60, reflecting a serum-level Th1-like response phenotype in calves. IL-10 levels remained persistently elevated from day 14 to 60, reflecting the regulatory capacity of IL-10 against pro-inflammatory immune responses. This sequential immune profile supports host defense against virulent M. bovis.

The clinical BRD diagnostic methods established by Love et al. and Maier et al. were used in this study to statistically analyze the BRD incidence in both groups. The BRD3 method is the simplest to implement, and diagnostic results obtained using this method were nearly identical to those of the industry-standard Wisconsin score [17]. Model 1 is the optimal weighted scoring model for BRD, specifically developed for weaned calves [18]. The results showed that the BRD incidence in the vaccine group was significantly lower than that in the control group. Furthermore, BCoV, *P. multocida*, and *M. bovis* were detected in all BRD calves in both groups, consistent with the pathogens detected by other researchers [44,45]. The detection rate of *M. bovis* in the control group reached as high as 91.67%, and the antibody positivity rates increased in this group, indicating the prevalence of *M. bovis* in this farm. The co-infections of *M. bovis* and other pathogens warrant close attention given that *M. bovis* is the major pathogen of BRD [4,46]. Previous studies have reported the synergistic co-infections of respiratory bacteria and viruses in BRD calves [1,19]. In this study, the BRD calves in the vaccine group showed a significantly lower infection rate of *M. bovis* compared with the control group. Notably, the infection rate of *P. multocida* was also significantly reduced. According to previously published evidence, one plausible hypothesis is that *M. bovis* damages the respiratory mucosal epithelium and impairs local mucosal immune defence, thereby creating favourable conditions for secondary colonization by *P. multocida* and other respiratory bacteria [7,24].

Regarding the protective effects against *M. bovis*-positive clinical BRD, the incidence of *M. bovis*-positive clinical BRD in the vaccine group was significantly lower than that in the control group. To quantitatively assess the actual field efficacy of the vaccine, this study adopted multiple epidemiological indicators. Vaccine efficacy (VE), which objectively evaluates the vaccine’s ability to protect against disease [47], was 61% in this trial. However, Brown pointed out that VE can be highly misleading, and the ARR and NNV should be preferred to assess the true protective efficacy of vaccines in randomized controlled clinical trials [20]. The ARR was 19.87% and NNV was approximately 5 in this study, meaning the incidence rate in unvaccinated herds was 19.87% higher than in vaccinated herds and that vaccination of approximately 5 calves could prevent one case. In epidemiological theory, a lower NNV value indicates higher intervention efficiency of vaccines, and the value obtained in this study was lower than that reported for some existing vaccines [14]. The above results can provide reference data for similar large-scale dairy farms. Simulated cost–benefit analysis was performed to explore the potential economic value of vaccination. The two-dose immunization cost was 20 Chinese yuan (CNY) per calf. To date, economic loss data for a single case of *M. bovis*-positive BRD have not been formally reported in Chinese domestic publications. Based on veterinary expert assessment, the total economic loss per case of *M. bovis*-positive BRD in calves was estimated at 400 CNY, covering expenses for diagnosis and treatment, veterinary medicines, labour input and production losses. Given an absolute risk reduction (ARR) of 19.87%, vaccination of 100 calves could prevent 19.87 cases of *M. bovis*-positive BRD. A BCR of 3.97 means that every 1 CNY invested in vaccination could bring approximately 3.97 CNY of returns via reduction of disease-related losses (BCR = Total avoided economic losses/Total vaccination investment = (19.87 × 400)/(100 × 20) = 3.97). The ICER was 100.65 CNY per prevented case of *M. bovis*-positive clinical BRD, meaning that an average vaccination cost of 100.65 CNY was required to prevent one case of *M. bovis*-positive BRD. These simulation results suggest that vaccination has potential economic advantages under the conditions of this dairy farm. Nevertheless, these calculations rely on veterinary expert assessment rather than individual-animal accounting records collected in this trial. Farm-specific real-world cost–benefit evaluation should be carried out in future investigations.

However, this study has limitations. First, the serological ELISA assay used in this work cannot distinguish antibodies induced by vaccination from those originating from natural field infection with *M. bovis*. Natural infection may lead to either overestimation or underestimation of VE, and this confounding factor cannot be ruled out. Second, nasal swabs for RT-qPCR pathogen detection were only collected from calves showing clinical BRD symptoms; hence, subclinical *M. bovis* colonization in vaccinated animals could not be monitored in this field trial. Third, delayed local adverse reactions were not screened in this trial, and hematological examinations and other safety-related assessments were not performed. In addition, the observation window for growth performance was relatively short. The long-term effects of the vaccine on calf growth remain unknown, and animals were not followed up to the breeding stage, making it impossible to evaluate subsequent reproductive performance. Future research should expand the number of experimental animals and farming scenarios, extend the observation period, and refine assessments of mucosal immune responses, artificial challenge studies, and production economics to comprehensively validate the scope of vaccine application, long-term immunoprotective efficacy, and field application value.

## 5. Conclusions

The inactivated *M. bovis* (strain HM) vaccine demonstrated a satisfactory tolerability, excellent immunogenicity, and satisfactory protective efficacy in the field. A vaccination protocol for 3–4-week-old Holstein calves, with a booster dose administered on day 14 after primary vaccination, significantly reduced the incidence of M. bovis-positive clinical BRD. These findings provide reliable field data and technical support for *M. bovis*-positive clinical BRD prevention and control on dairy farms. Nevertheless, these findings should be further validated across diverse dairy production systems.

## Figures and Tables

**Figure 1:**
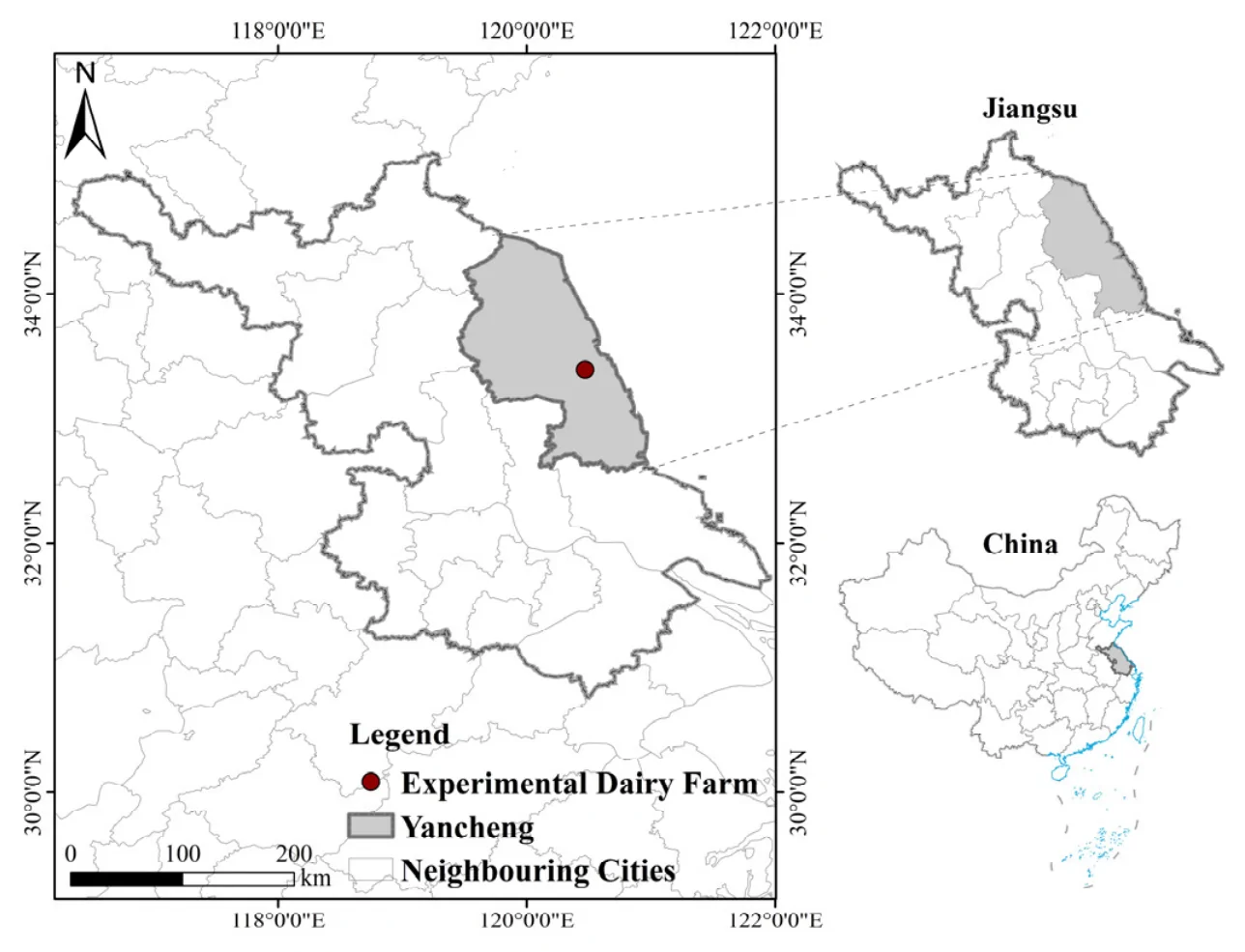
Geographical location of the dairy farm.

**Figure 2:**
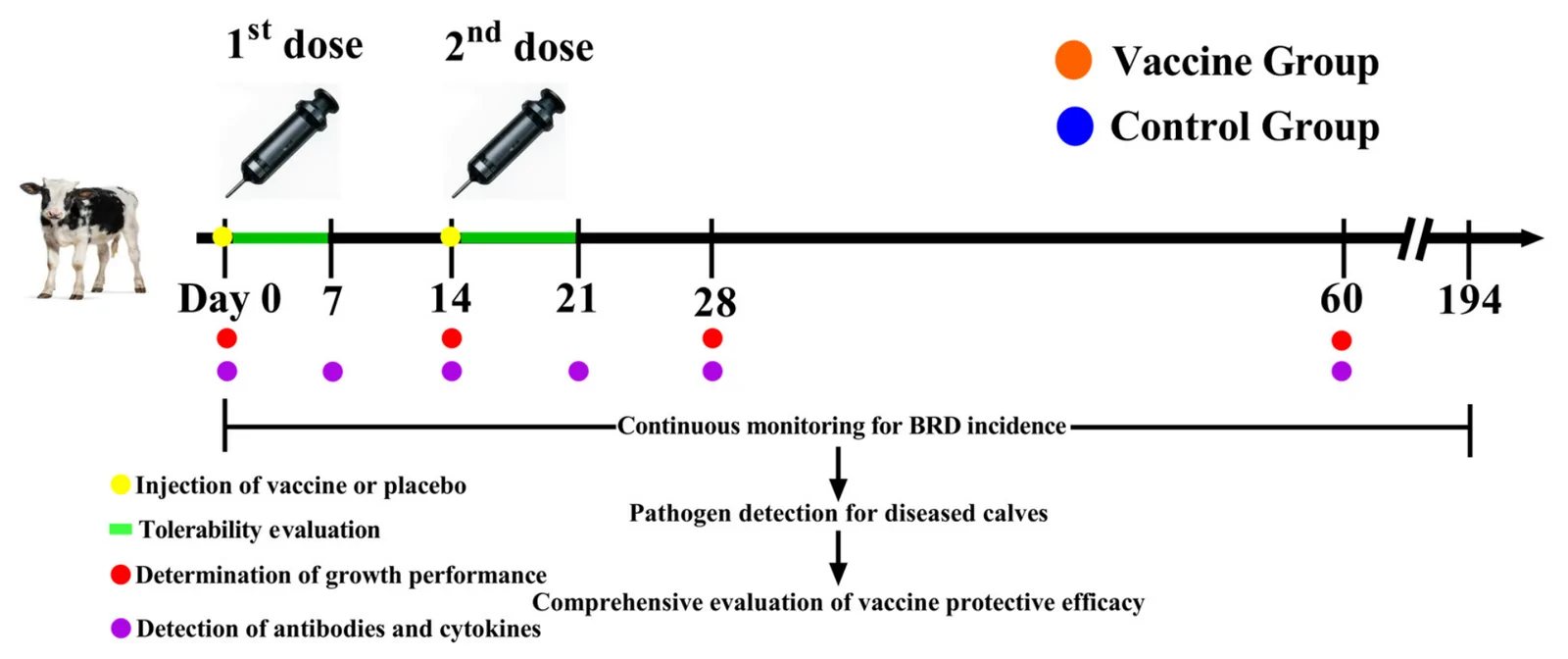
Schematic diagram of the study design.

**Figure 3:**
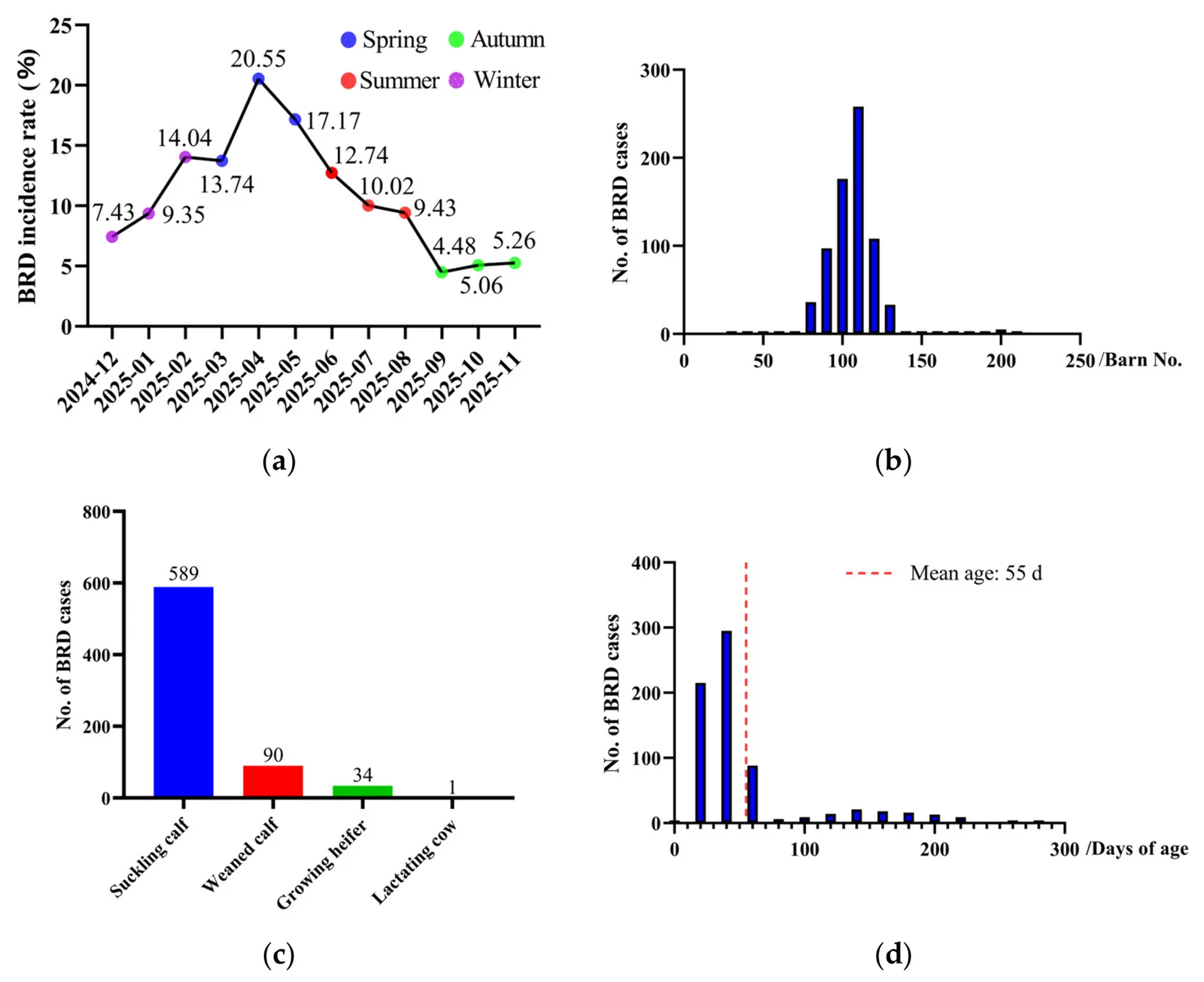

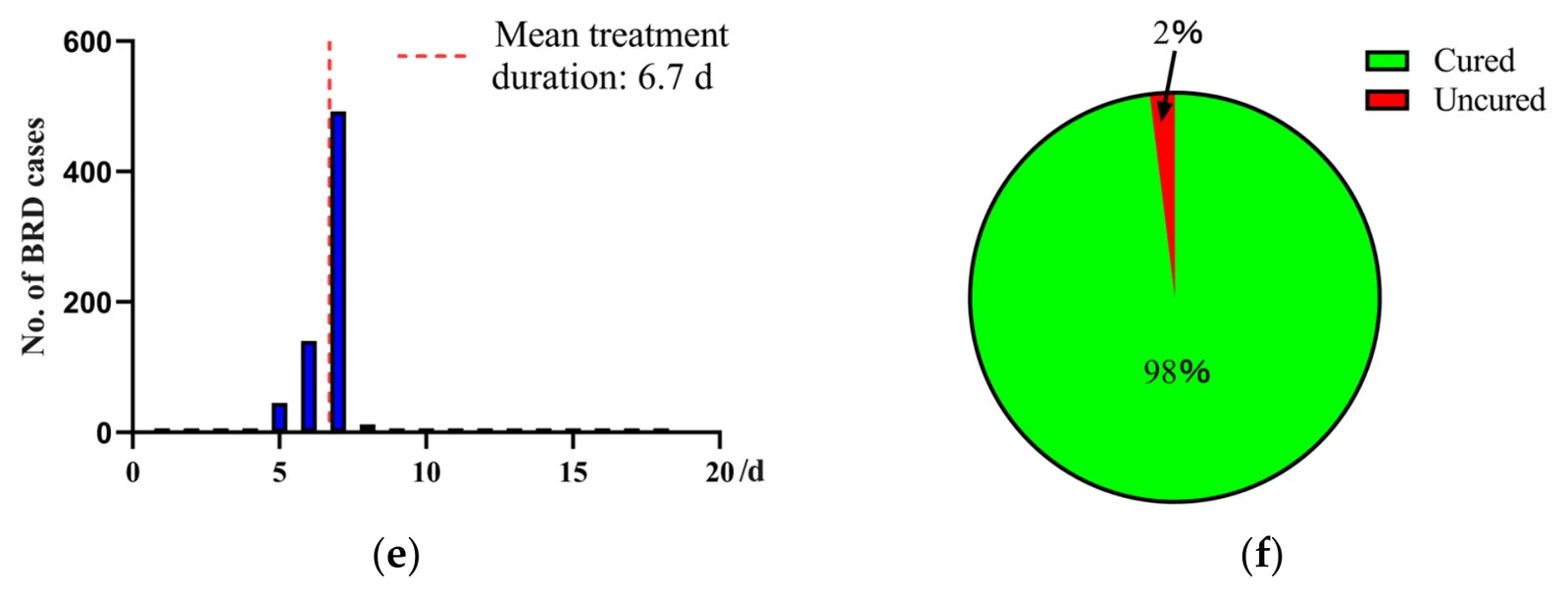
Epidemiological analysis of BRD. (**a**) Line chart of temporal distribution. (**b**) Histogram of BRD case frequency by barn. (**c**) Bar chart of BRD cases across different physiological stages. (**d**) Histogram of case frequency by age (≤365 days). (**e**) Histogram of case frequency by treatment duration (≤30 days). (**f**) Pie chart of case outcomes.

**Figure 4:**
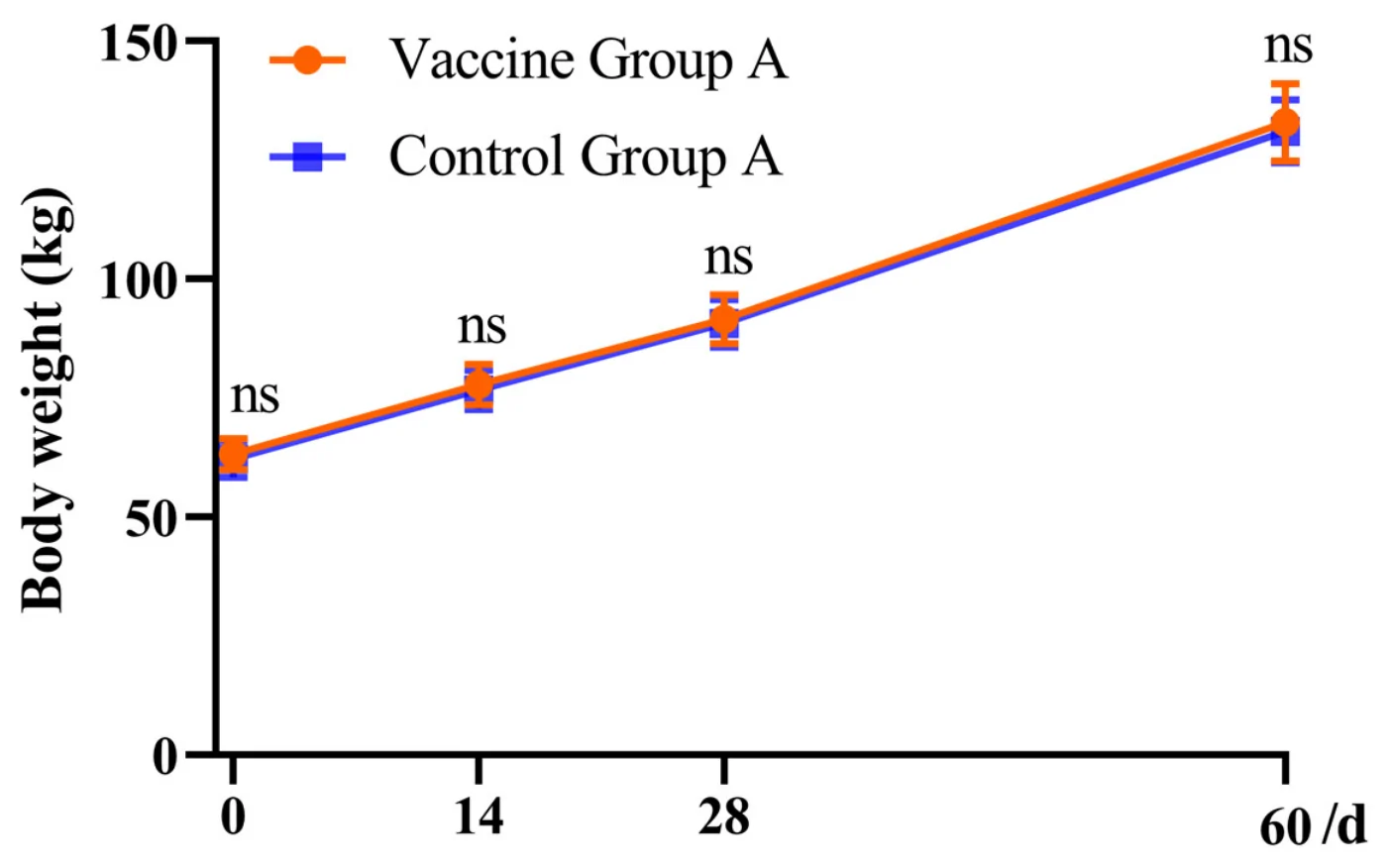
Changes in body weight at different time points in subgroup A. All data are presented as mean ± SD (n = 16); ns: *p* > 0.05.

**Figure 5:**
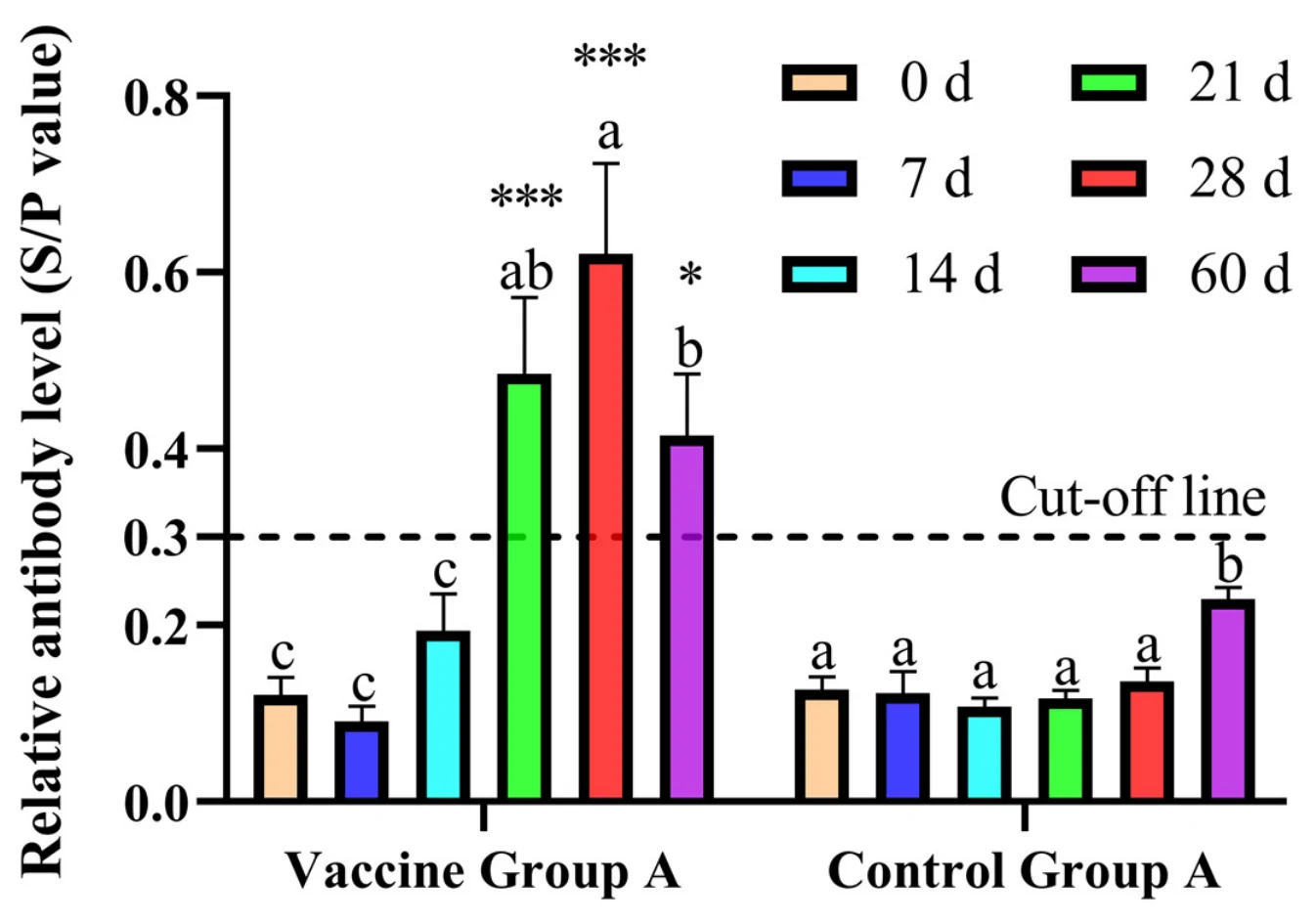
Changes in relative antibody levels (S/P values) at different time points. All data are presented as mean ± SD (n = 16); for comparisons at different time points within the same group, bars marked with different lowercase letters indicate *p* < 0.05, while those with identical lowercase letters indicate *p* > 0.05. For intergroup comparisons at the same time point, *: *p* < 0.05, ***: *p* < 0.001.

**Figure 6:**
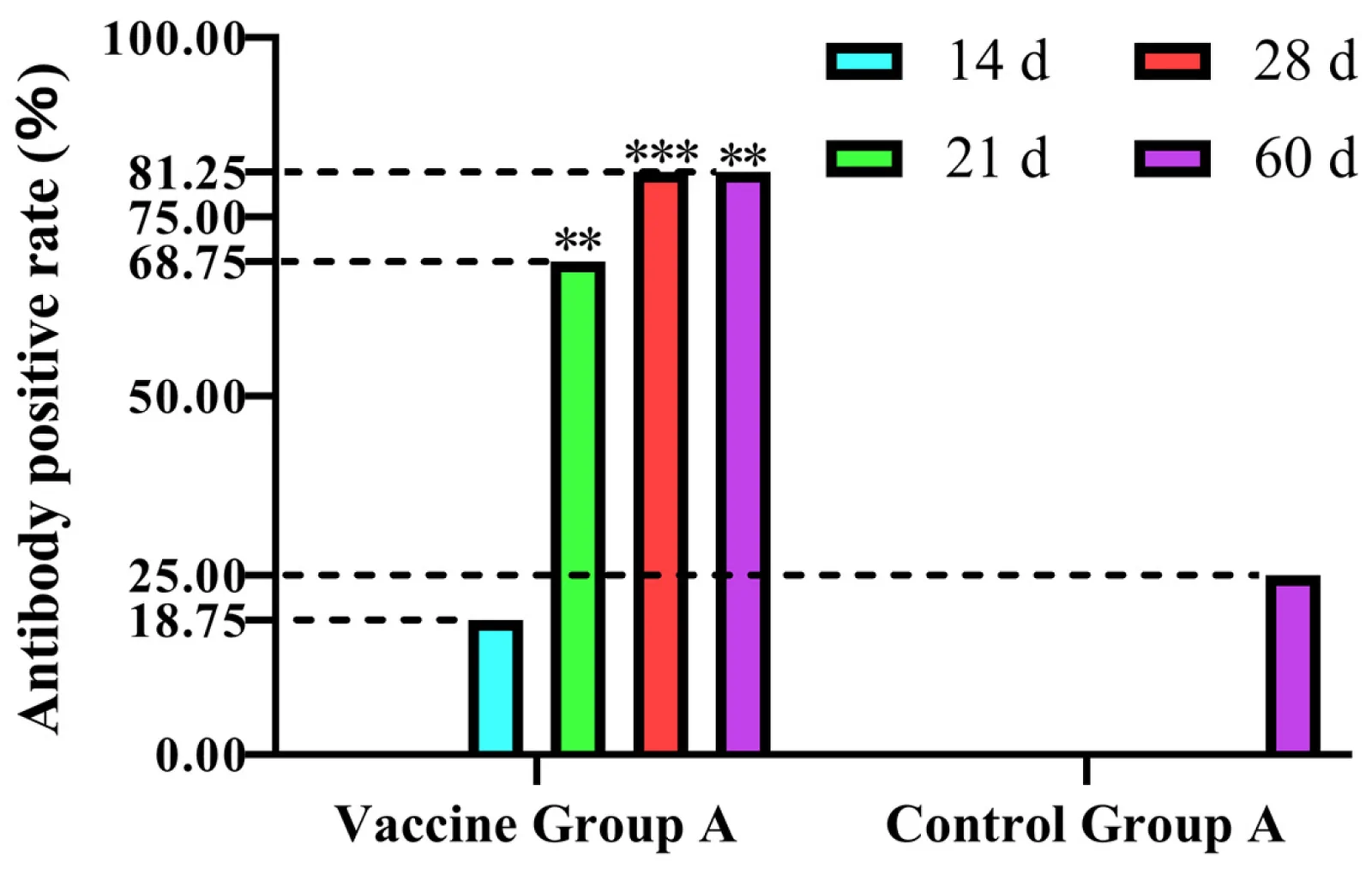
Changes in antibody seropositivity rates at different time points. **: *p* < 0.01, ***: *p* < 0.001.

**Figure 7:**
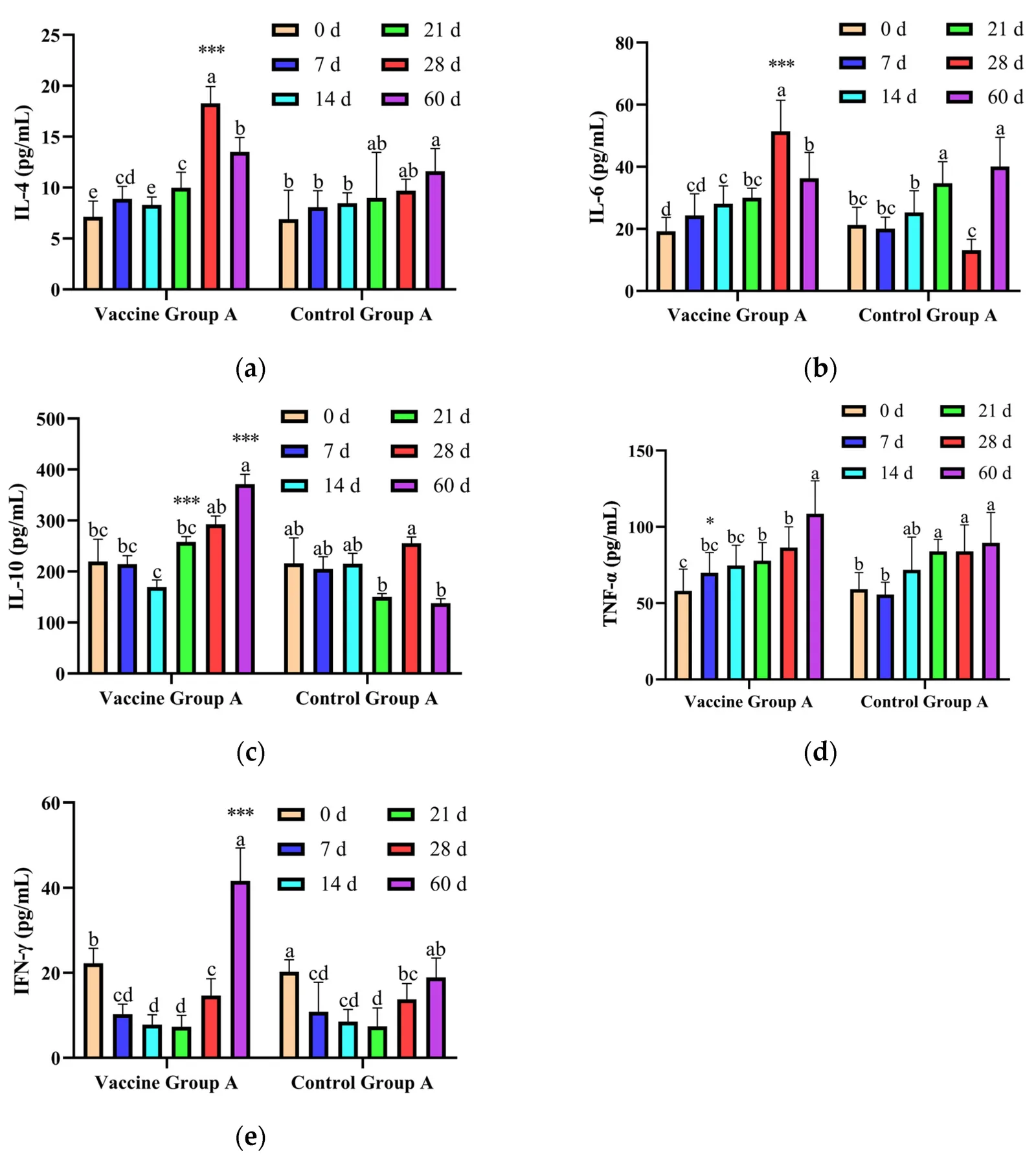
Changes in serum cytokine levels at different time points. (**a**) IL-4. (**b**) IL-6. (**c**) IL-10. (**d**) TNF-α. (**e**) IFN-γ. All data are presented as mean ± SD (n = 16). All data are presented as mean ± SD (n = 16); for comparisons at different time points within the same group, bars marked with different lowercase letters indicate *p* < 0.05, while those with identical lowercase letters indicate *p* > 0.05. For intergroup comparisons at the same time point, *: *p* < 0.05, ***: *p* < 0.001.

**Figure 8:**
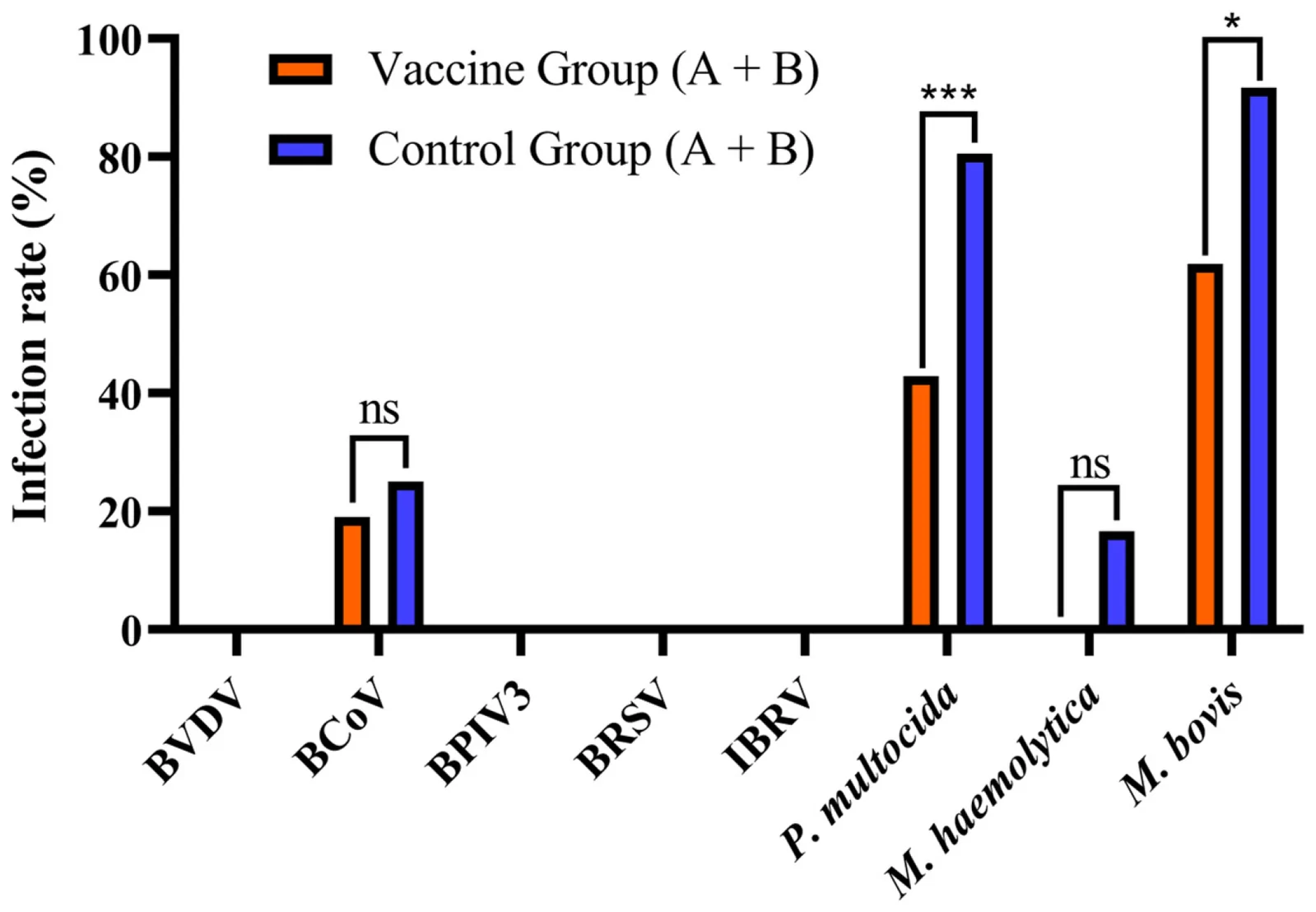
Infection rates of respiratory pathogens in BRD calves of vaccine and control groups (A + B). ns: *p* > 0.05, *: *p* < 0.05, ***: *p* < 0.001.

**Table 1:** The fluorescence RT-qPCR reaction system.

Reagent Volume (μL)	Volume of Extracted Nucleic Acid (μL)	Total Reaction System (μL)
8-plex Reaction Mix 1 (20)	5	25
8-plex Reaction Mix 2 (20)	5	25
8-plex Reaction Mix 3 (20)	5	25
Negative control	A set of negative and positive controls shall be prepared for each reaction mix; each control system contains 20 μL reaction mix plus 5 μL negative/positive template, respectively.	
Positive control		

8-plex Reaction Mix 1 targets bovine viral diarrhea virus (BVDV) and bovine coronavirus (BCoV); 8-plex Reaction Mix 2 targets bovine parainfluenza virus type 3 (BPIV3), bovine respiratory syncytial virus (BRSV) and infectious bovine rhinotracheitis virus (IBRV); 8-plex Reaction Mix 3 targets *Pasteurella multocida* (*P. multocida*), *Mannheimia haemolytica* (*M. haemolytica*) and *M. bovis*.

**Table 2:** Incidence of BRD on the dairy farm.

Period	No. of Cattle	No. of BRD Cases	Overall Incidence (%)	No. of BRD Cases by Season			
				Spring	Summer	Autumn	Winter
December 2024–November 2025	7702	714	9.27	237	130	136	211

**Table 3:** Statistical data of abnormal clinical signs in calves post immunization.

Observation Category	Detected Items	No. of Animals with Abnormal Signs			
		7 days after primary vaccination		7 days after booster vaccination	
		Vaccine group (A + B)	Control group (A + B)	Vaccine group (A + B)	Control group (A + B)
Systemic reactions	Depression	0	0	0	0
	Reduced feed intake	0	0	0	0
	Hyperthermia (>39.5 °C)	0	0	0	0
	Stress response	0	0	0	0
	Paralysis	0	0	0	0
	Anaphylactic shock	0	0	0	0
	Death	0	0	0	0
	Diarrhea	0	0	0	0
	Cough	0	0	0	0
	Nasal discharge	0	0	0	0
Injection site reactions	Swelling and erythema	0	0	0	0
	Induration	0	0	0	0
	Abscessation/inflammation	0	0	0	0

**Table 4:** Comparison of body weight and ADG of calves between vaccine group A and Control group A.

Item	Time (d)	Vaccine Group A	Control Group A	*p*-Value
Body weight (kg)	0	63.15 ± 3.28	62.15 ± 3.91	0.440
	14	77.78 ± 4.26	76.65 ± 4.16	0.456
	28	91.54 ± 5.19	90.62 ± 5.05	0.623
	60	132.96 ± 8.09	131.09 ± 6.60	0.485
ADG (g/d)	0–14	1044.64 ± 162.09	1035.71 ± 113.68	0.858
	0–28	1010.95 ± 125.99	1016.74 ± 122.24	0.898
	0–60	1162.22 ± 113.38	1148.95 ± 93.18	0.724
	14–28	974.28 ± 170.22	997.76 ± 211.27	0.737
	14–60	1197.10 ± 139.92	1183.42 ± 119.93	0.767
	28–60	1213.67 ± 360.93	1264.64 ± 128.40	0.598

All data are presented as mean ± SD (n = 16).

**Table 5:** Statistics on BRD incidence in the vaccine group and control group.

Period	Time (d)	Vaccine Group (A + B)	Control Group (A + B)	*p*-Value
		Incidence rate of BRD (%)		
Pre-weaning	0–60	9.09 (9/99)	16.00 (16/100)	0.141
Post-weaning	61–120	8.08 (8/99)	11.00 (11/100)	0.484
	121–194	4.06 (4/99)	9.00 (9/100)	0.157
	0–194	21.21 (21/99)	36.00 (36/100)	0.021
		BRD case fatality rate (%)		
	0–194	0	0	

**Table 6:** Statistics detection results of common pathogens in the vaccine group and control group.

Pathogen	Vaccine Group (A + B)		Control Group (A + B)		*p*-Value
	Positive number	Infection rate (%)	Positive number	Infection rate (%)	
BVDV	0	0	0	0	
BCoV	4	19.05 (4/21)	9	25.00 (9/36)	0.605
BPIV3	0	0	0	0	
BRSV	0	0	0	0	
IBRV	0	0	0	0	
*P. multocida*	9	42.86 (9/21)	29	80.56 (29/36)	0.004
*M. haemolytica*	0	0	6	16.66 (6/36)	0.075
*M. bovis*	13	61.90 (13/21)	33	91.67 (33/36)	0.012

**Table 7:** Statistics on *M. bovis*-positive clinical BRD incidence in the vaccine group and control group.

Period	Time (d)	Vaccine Group (A + B)	Control Group (A + B)	*p*-Value
Pre-weaning	0–60	5.05 (5/99)	9.00 (9/100)	0.276
Post-weaning	61–120	4.04 (4/99)	11.00 (11/100)	0.063
	121–194	4.04 (4/99)	13.00 (13/100)	0.024
	0–194	13.13 (13/99)	33.00 (33/100)	<0.001

**Table 8:** Statistical analysis of vaccine protective efficacy.

Item	Value
RR	0.39
VE (%)	61
ARR (%)	19.87
NNV	5.03

## Data Availability

The data presented in this study are available on request from the corresponding author. The data are not publicly available due to commercial confidentiality agreements with the cooperating farm.

## Abbreviations

The following abbreviations are used in this manuscript:

BRD	Bovine Respiratory Disease
RR	Relative risk
VE	Vaccine efficacy
ARR	Absolute risk reduction
NNV	Number needed to vaccinate
BVDV	Bovine Viral Diarrhea Virus
BCoV	Bovine Coronavirus
BPIV3	Bovine Parainfluenza Virus Type 3
BRSV	Bovine Respiratory Syncytial Virus
IBRV	Infectious Bovine Rhinotracheitis Virus
*P. multocida*	*Pasteurella multocida*
*M. haemolytica*	*Mannheimia haemolytica*
*M. bovis*	*Mycoplasma bovis*
IL-4	Interleukin-4
IL-6	Interleukin-6
IL-10	Interleukin-10
TNF-α	Tumor necrosis factor-α
IFN-γ	Interferon-γ
RT-qPCR	Reverse transcription quantitative polymerase chain reaction
SD	Standard deviation
S/P	Sample-to-positive ratio
ADG	Average daily gain
CNY	Chinese yuan
